# The aetiology and trajectory of anabolic-androgenic steroid use initiation: a systematic review and synthesis of qualitative research

**DOI:** 10.1186/1747-597X-9-27

**Published:** 2014-07-02

**Authors:** Dominic Sagoe, Cecilie Schou Andreassen, Ståle Pallesen

**Affiliations:** 1 Department of Psychosocial Science, University of Bergen, Christiesgate 12, 5015 Bergen, Norway; 2 The Competence Centre, Bergen Clinics Foundation, Bergen, Norway

**Keywords:** Anabolic-androgenic steroids, Metasynthesis, Narrative synthesis, Systematic review, Aetiology, Trajectory, Qualitative research, Interview

## Abstract

**Background:**

To our knowledge, there has never been a systematic review and synthesis of the qualitative literature on the trajectory and aetiology of nonmedical anabolic-androgenic steroid (AAS) use.

**Methods:**

We systematically reviewed and synthesized qualitative literature gathered from searches in PsycINFO, PubMed, ISI Web of Science, Google Scholar, and reference lists of relevant literature to investigate AAS users’ ages of first use and source(s), history prior to use, and motives/drives for initiating use. We adhered to the recommendations of the UK Economic and Social Research Council’s qualitative research synthesis manual and the PRISMA guidelines.

**Results:**

A total of 44 studies published between 1980 and 2014 were included in the synthesis. Studies originated from 11 countries: the United States (*n =* 18), England (*n =* 8), Australia (*n =* 4), Sweden (*n =* 4), both England and Wales (*n =* 2), and Scotland (*n =* 2). One study each originated from Brazil, Bulgaria, Canada, France, Great Britain, and Norway. The majority of AAS users initiated use before age 30. Sports participation (particularly power sports), negative body image, and psychological disorders such as depression preceded initiation of AAS use for most users. Sources of first AAS were mainly users’ immediate social networks and the illicit market. Enhanced sports performance, appearance, and muscle/strength were the paramount motives for AAS use initiation.

**Conclusions:**

Our findings elucidate the significance of psychosocial factors in AAS use initiation. The proliferation of AAS on the illicit market and social networks demands better ways of dealing with the global public health problem of AAS use.

## Background

Several qualitative investigations have sought to understand the aetiology and trajectory of nonmedical AAS use initiation. However, to our knowledge, there has never been a systematic review and synthesis of the qualitative literature on this important area of nonmedical AAS use. An investigation of this type is important because a global perspective of nonmedical AAS use initiation is necessary for the understanding of this global public health problem [1].

A review and synthesis of the qualitative research on AAS use initiation is also important in light of expressed concern regarding the validity and reliability of survey research on AAS use [2]. Moreover, it has been suggested that the failure of health practitioners and public health officials to appreciate people’s perception of antecedents and risk factors is a major hindrance to the success of public health interventions [3,4]. Hence, data on initiation and trajectories of AAS use are important for prevention purposes.

We carried out, as far as we are aware, the pioneering systematic review and synthesis of the qualitative studies presenting data on the initiation of nonmedical AAS use. The United Kingdom’s Economic and Social Research Council’s manual on the synthesis of qualitative literature [5] indorses the formulation of research questions or hypothesis prior to synthesis. The research questions guiding the present study were: (a) at what age(s) do AAS users have their debut?, (b) what are the psychosocial histories of AAS users prior to the initiation of AAS use?, (c) what are the sources of AAS users’ first AAS?, and (d) what are the motives and drives for initiating AAS use?

## Method

### Search strategy and inclusion criteria

We conducted a comprehensive literature search in PsycINFO, PubMed, ISI Web of Science, and Google Scholar. The following keywords: ‘anabolic steroid’, ‘doping’, and ‘performance enhancing drug’, were each used in combination with ‘interview’, ‘focus group’, and ‘qualitative’ for searches in PubMed and ISI Web of Science. Due to unusually high superfluous returns from the above permutation of keywords, ‘anabolic steroid + doping + performance enhancing drug + interview + focus group + qualitative’ was used in searches in PsycINFO and Google Scholar. The literature search was completed in June 2014. From an initial pool of 10,106 hits, 7,720 articles were evaluated after removing duplicates. In addition, a manual check of reference lists of identified studies was conducted in search of potential unidentified studies. Searches were also conducted in online databases and websites. We identified 4 new articles through this grey literature search. Thus, a total of 7,724 were settled on after eliminating duplicates. After evaluating the 7,724 papers based on titles and abstracts, 95 full-text papers were retrieved for screening.

**Figure 1:**
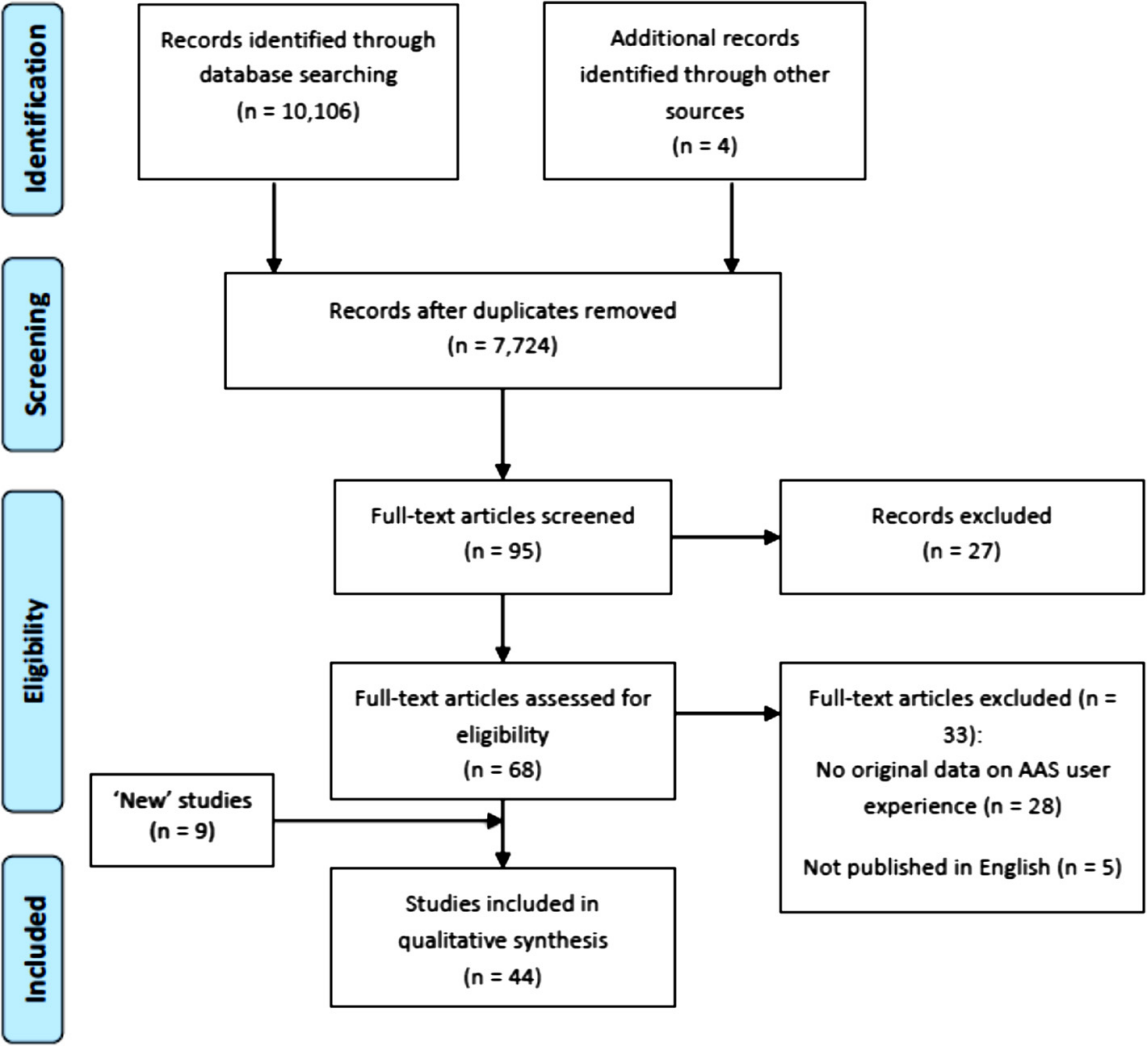
Flow diagram of systematic literature search.

After initial screening of the 95 full-text papers, 68 papers were identified. Of the 68 papers scrutinized, 35 studies met the following key criteria for inclusion: (a) studies presented original information on the experiences of AAS users (b) studies employed qualitative approaches in data collection (interviews, focus groups, or case studies) and presentation of results, and (c) studies were published in English. Four recent studies [6-9] and five others [10-14] were later discovered and included in the analysis. We again inspected the characteristics of extracted studies for similarities to curb duplicate extraction and synthesis. Thus, a total of 44 articles were included in the analysis. The literature search strategy adhered to Shaw et al.’s [15] recommendations for finding qualitative research as well as the Preferred Reporting Items for Systematic Reviews and Meta-Analyses (PRISMA) guidelines [16]. Figure 1 presents the process of the search and selection of relevant studies according to the PRISMA guidelines.

### Data extraction and synthesis

The first author scrutinized and selected studies. Smith et al.’s [17] Interpretative Phenomenological Analysis (IPA) was used to analyse the studies because it facilitates in-depth exploration of the meanings of experiences [18]. Each full-text paper was regarded as a transcript. The first author read through the full-text papers several times, gaining an overall sense of the themes in the studies through this process. These themes were then highlighted. We developed a standardized data extraction form unto which the first author and another reviewer independently extracted the following data from the included studies: author name and publication year, country, study type, type of AAS users involved in the study, and recruitment site or mode. These characteristics are presented in Table 1. The first author independently coded the full-text papers according to the presence or absence of the following themes: (a) age(s) of first use, (b) history prior to use, (c) source(s) of first AAS, and (d) motive(s)/drive(s) for initiating use. These characteristics are presented in Table 2. Statistical inferences have little meaning in qualitative synthesis. However, the presence of a theme in multiple studies may be evidence of the validity of the theme [4]. In this regard, we have presented all the studies that fall under each theme.

**Table 1:** Qualitative studies presenting data on AAS use initiation

**First author, year, reference**	**Country/countries**	**Study type**	**AAS user(s)**	**Recruitment site(s)/mode**
Annitto 1980 [19]	USA	Interview	17-year-old male bodybuilder	Clinic
Bardick 2006 [20]	Canada	Interview	8 male weightlifters aged 21 to 35 years	Gymnasium
Bilard 2011 [21]	France	Interview	203 bodybuilders	Voluntary
Boyadjiev 2000 [22]	Bulgaria	Case study	20-year-old male bodybuilder	Clinic
Copeland 2000 [23] and Peters 1997 [24]	Australia	Interview and questionnaire^†^	100 persons (6 female) aged 18 to 50 years	Gymnasium, sports shops and associations, syringe exchange centre, radio interviews, advertisements
Cornford 2014 [9]	England	Interview and focus group	30 males aged 20 to 40 years	Syringe exchange centre
Fudala 2003 [25]	USA	Interview	7 males aged 22 to 33 years	Gymnasium and community
Grogan 2006 [26]	England	Interview	11 bodybuilders (6 female) aged 20 to 39 years	Gymnasium
Gruber 1999 [27]	USA	Interview	5 female bodybuilders	Gymnasium
Hegazy 2013 [28]	USA	Case study	28-year-old male	Clinic
Joubert 2014 [7]	England	Interview	6 males aged 26 to 42 years	Addiction charity
Katz 1990 [29]	USA	Case study	23-year-old male bodybuilder	Gymnasium
Khorrami 2002 [30]	USA	Interview	2 male weightlifters aged 24 and 29 years	Voluntary
Kimergård 2014 [6,8]	England and Wales	Interview	24 males aged 21 to 61 years; mean age 34 years	Gymnasium, prison, steroid clinic and charity, syringe exchange centre
Klötz 2010 [31]	Sweden	Interview	33 male prisoners aged 21 to 52 years	Prison
Korkia 1993 [12]	England, Scotland, and Wales	Interview	110 persons (13 female) aged 16 to 63 years	Gymnasium, clinic, syringe exchange centre
Korkia 1996 [13]	England	Interview and questionnaire^†^	15 females; mean age 28 years	Not specified
Kusserow 1990 [32]	USA	Interview	72 (6 female) persons (mostly adolescents); 14 to 25 years; mean age 20 years	Not specified
Malone 1995 [33]	USA	Interview	77 (6 female) powerlifters and bodybuilders	Gymnasium
Maycock 2005 [34], 2007 [35]	Australia	Interview	42 males	Gymnasium, night club, community
McKillop 1987 [36]	Scotland	Interview	8 male bodybuilders aged 17 to 32 years	Gymnasium
Midgley 1999 [37]	England	Interview and questionnaire^†^	50 male bodybuilders and weight trainers aged 17 to 46 years	Gymnasium and syringe exchange centre
Nøkleby 2013 [38]	Norway	Interview	9 male drug users aged 22 to 35 years	Clinic
O’Sullivan 2000 [39]	Australia	Interview	41 males aged 16 to 36 years	Clinic
Olrich 1999 [40]	USA	Interview	10 male weightlifters; 9 aged 18 to 35 years, 1 aged 57 years	Gymnasium
Pappa 2012 [41]	England	Interview	9 athletes aged 19 to 26 years	Community via snowball sampling
Petrocelli 2008 [42]	USA	Interview	37 male gym users aged 19 to 43 years	Gymnasium
Pope 1990 [43]	USA	Interview	3 male arrested weightlifters aged 23, 24, and 32 years	Justice system
Pope 1993 [44]	USA	Interview	55 bodybuilders; mean age 28 years; 3 bodybuilders; 19 years, 26 years, 27 years	Gymnasium
Pope 1996 [45]	USA	Case study	16-year-old male	Clinic
Pope 1996 [45]	USA	Interview	9 male prisoners	Prison
Rashid 2000 [14]	USA	Case study	40-year-old male	Clinic
Schwingel 2012 [46]	Brazil	Interview	147 male power sportspeople aged 18 to 42 years	Exercise laboratory
Scull 2013 [47]	USA	Interview	7 male strippers	Strip club
Skårberg 2007 [48]	Sweden	Interview and questionnaire^†^	18 male drug users; mean age 35 years	Clinic
Skårberg 2008 [49]	Sweden	Interview	6 drug users (2 female)	Clinic
Skårberg 2009 [50] and 2007 [48]	Sweden	Interview and questionnaire^†^	32 male drug users 18 male drug users; mean age 35 years	Clinic
Tallon 2007 [11]	Scotland	Interview and questionnaire^†^	30 males aged 18 to 43 years; mean age 27 years	Gymnasium
Todd 1987 [51]	USA	Interview	2 persons (27-year-old female weightlifter; 1 former male NFL player)	Not specified
Vassalo 2010 [52]	USA	Interview	39 male athletes aged 18 to 35 years	Acquaintances
Walker 2011 [10]	England	Interview and questionnaire^†^	41 males; 20 to 30 years (majority)	Syringe exchange centre
Wilson-Fearon 1999 [53]	England	Case study	29-year-old bodybuilder	Not specified

^†^We relied on the qualitative results generated from the interview.

**Table 2:** Characteristics of qualitative studies presenting data on AAS use initiation

**First author, year, reference**	**Initiation age(s)**	**History prior to use**	**Source(s)**	**Motive(s)/drive(s) for use**
Annitto 1980 [19]	16 years	Weightlifting	Illicit market	Appearance
Bardick 2006 [20]	Not specified	Weight training	Not specified	Appearance, confidence, media, personal security, psychological well-being, sports
Bilard 2011 [21]	Not specified	Bodybuilding	Friends, dealers, others, relatives, teammates	Appearance, muscle, physiological recovery, psychological, sports, sports norm, other
Boyadjiev 2000 [22]	19 years	Bodybuilding, cycling	Not specified	Sports
Copeland 2000 [23] and Peters 1997 [24]	14 to 46 years; mean 25 years	Not specified	Coaches/trainers, dealers, doctors, friends, gym employees, other, pharmacists, mail order relatives, veterinarians	Appearance, muscle, other, physiological recovery/injury prevention, sports
Cornford 2014 [9]	≤ 30 years (n = 14)	Not specified	Not specified	Muscle, personal security, physiological recovery, sports
Fudala 2003 [25]	≤ 26 years	Negative body image, poor self-esteem, psychological disorders, troubled background	Not specified	Appearance, psychological
Fudala 2003 [25]	≤ 31 years	Negative body image, low self-efficacy, troubled background	Not specified	Appearance, muscle
Fudala 2003 [25]	17 years	Football	Relative	Appearance, muscle, sports
Fudala 2003 [25]	26 years	Troubled background	Not specified	Appearance, muscle
Fudala 2003 [25]	21 years	Binge eating, psychological disorders, troubled background	Not specified	Not specified
Fudala 2003 [25]	27 years	Troubled background, weightlifting	Friend	Sports
Fudala 2003 [25]	24 years	Bodybuilding	Not specified	Sports
Grogan 2006 [26]	15 years, 16 years, 18 years, 19 years, 20 years, 21 years, 23 years, 29 years	Bodybuilding	Not specified	Appearance, media, occupational, sports, sport/social norm
Gruber 1999 [27]	Not specified	Polydrug use, psychological disorders, troubled background	Trainer	Appearance, muscle, personal security
Hegazy 2013 [28]	22 years	Polydrug use, psychological disorders, troubled background	Friends	Appearance, muscle, recovery
Joubert 2014 [7]	16 to 24 years	Low self-esteem, negative body image, troubled background	Not specified	Appearance, confidence, family influence, muscle, peer influence, personal security, psychological well-being, self-esteem, social pressure
Katz 1990 [29]	21 years	Bodybuilding	Not specified	Sports
Khorrami 2002 [30]	Not specified	Football, negative body image, weightlifting	Gym employee	Appearance, family influence, muscle, sports
Kimergård 2014 [6,8]	16 years; mean age 25 years	Not specified	Not specified	Appearance, muscle, occupational, sports
Klötz 2010 [31]	Not specified	Not specified	Not specified	Aggression, appearance, muscle, other, psychological, sports, sport/social norm
Korkia 1993 [12]	16 years, 18 years, 32 years, 54 years	Weight training	Coach, dealers, doctors, friends/teammates, gym owner/employee	Muscle, physiological recovery, sports
Korkia 1996 [13]	19 years, 23 years	Not specified	Friends, gym owners/employees, husbands/boyfriends	Muscle, sports
Kusserow 1990 [32]	14 years, 15 years, 17 years, 18 years, ≤ 25 years^†^	Football, bodybuilding, negative body image, polydrug use	Coach/team doctor, dealers, doctors, friends/teammates, gym employees, pharmacists, veterinarians	Aggression, sports scholarship, appearance, coaches’ approval, famous athletes, media influence, parental approval, peer influence, sexual attraction, sports
Malone 1995 [33]	24 years	Weightlifting	Not specified	Appearance, muscle, injury prevention/recovery, sports, sport norm
Maycock 2005 [34] and 2007 [35]	24 years, 25 years	Complacent trainers, negative body image, weight training	Dealers	Appearance, aggression, coaches’ approval, peer influence, sexual attraction, sports
McKillop 1987 [36]	Not specified	Not specified	Not specified	Aggression, injury prevention/recovery, muscle, sports
Midgley 1999 [37]	Not specified	Not specified	Not specified	Appearance, injury prevention/recovery, psychological well-being, muscle, peer influence, sports, sexual attraction
Nøkleby 2013 [38]	Not specified	Other drug use, sports/exercise	Friend	Appearance, muscle, psychological well-being, sports
O’Sullivan 2000 [39]	Not specified	Not specified	Friends, gym dealers, medical practitioners	Appearance, muscle
Olrich 1999 [40]	23 years	Bodybuilding	Not specified	Appearance, curiosity, occupational, peer influence, psychological well-being, social/sexual attraction, sports, sport/social norm
Pappa 2012 [41]	Not specified	Athletics	Not specified	Appearance, concentration, curiosity, muscle, social influence, sports, sport norm
Petrocelli 2008 [42]	Not specified	Long-term exposure to muscle magazines, negative body image, weight training	Dealer, friend, external internet, gym dealer	appearance, confidence, muscle, psychological well-being, sexual attraction
Pope 1990 [43]	30 years	Weightlifting	Not specified	Not specified
Pope 1990 [43]	21 years	Weightlifting	Not specified	Sports
Pope 1990 [43]	20 years	Weightlifting	Not specified	Sports
Pope 1993 [44]	19 years	Anorexia nervosa, negative body image, psychological disorders, weightlifting	Not specified	Appearance
Pope 1993 [44]	18 years	Anorexia nervosa, negative body image, weightlifting	Not specified	Appearance
Pope 1993 [44]	24 years	Anorexia nervosa, negative body image, weightlifting	Not specified	Appearance
Pope 1996 [45]	14 years	Psychological disorders, weightlifting	Not specified	Appearance, confidence, muscle, psychological
Rashid 2000 [14]	38 years	Psychological disorders, other drug use, troubled background	Not specified	Appearance, confidence, muscle, psychological
Schwingel 2012 [46]	Not specified	Not specified	Friends, illicit market	Appearance, muscle, occupational, sport
Scull 2013 [47]	18 years	Male stripping	Not specified	Appearance, muscle, occupational
Skårberg 2008 [49]	20 years	Troubled background, weight training	Friend	Appearance, muscle
Skårberg 2008 [49]	21 years	Troubled background, weight training	Friend	Muscle
Skårberg 2008 [49]	16 years	Irritability, troubled background, weight training	Not specified	Curiosity, muscle
Skårberg 2008 [49]	20 years	Bodybuilding, other sports	Not specified	Appearance, sports, sport norm
Skårberg 2008 [49]	20 years	Bodybuilding, troubled background,	Not specified	Sports, sport norm
Skårberg 2008 [49]	21 years	Other sports, troubled background, weight training	Intimate partner	Appearance, muscle
Skårberg 2009 [50] and 2007 [48]	15 to 28 years	Troubled background	Not specified	Appearance, muscle, sports
Tallon 2007 [11]	18 to 43 years	Weight training, other sports	Friends/training partners	Appearance, confidence, injury/illness prevention, muscle, psychological, sexual attraction
Todd 1987 [51]	Not specified	Powerlifting	Dealer	Sports, sport norm
Vassalo 2010 [52]	Not specified	Football	Not specified	Sports scholarship
Walker 2011 [10]	20 to 30 years^†^	Not specified	Gym dealer	Appearance, muscle
Wilson-Fearon 1999 [53]	Not specified	Bodybuilding	Not specified	Sports

^†^Majority.

### Quality of extraction, included studies, and synthesis

To assess the quality of the extraction, we calculated inter-reviewer reliability for the two reviewers in SPSS version 20 (IBM Corp.) [54]. Sensitivity analysis is conducted in the synthesis of qualitative research to examine the effect of the exclusion of high or poor quality studies on the overall findings. We assessed the relevance of the included papers according to the four themes: (a) age(s) of first use, (b) history prior to use, (c) source(s) of first AAS, and (d) motivation(s) for use (see Table 2). Each theme was scored ‘1’ thus yielding a possible total score of ‘4’. Subsequently, we excluded studies that scored ≤ 2 out of 4 on the themes and investigated the effect of the exclusion on our synthesis and results. Moreover, as most of the included studies were conducted in the United States, we excluded the United States studies to investigate the effect of the exclusion on the quality of our synthesis and results.

## Results and discussion

### Strength of extraction, included studies, and synthesis

The inter-reviewer reliability for the reviewers was found to be Kappa = 0.82 (*p* < 0.001) indicating very good agreement between the two reviewers [55]. Consensus was reached on discrepant extractions through further review and discussion. Thirty-eight (38) of the 44 studies scored ≥ 3 out of 4 on the themes and were thus deemed to be of high relevance. Six studies [31,36,37,41,46,52] scored ≤ 2 out of 4 on relevance and were therefore excluded in the quality analysis. However, when we removed the study characteristics generated from these studies in the sensitivity analysis, our themes or results did not change. Consequently, we retained them in the final analysis. Similarly, the removal of the study characteristics generated from the studies originating from the United States did not affect the quality of our themes or results. Thus, they were also retained in the final analysis. The sensitivity analysis therefore indicated a strong synthesis of included studies.

### Description of studies

A total of 44 studies were included in the metasynthesis. Participants’ ages ranged from 14 to 63 years. The year of publication of the studies ranged from 1980 [19] to 2014 [6-9,11]. Studies originated from 11 countries although most originated from the United States (*n =* 18), followed by England (*n =* 8), Australia (*n =* 4), Sweden (*n =* 4), both England and Wales (*n =* 2), and Scotland (*n =* 2). Moreover, one study each originated from Brazil, Bulgaria, Canada, France, Great Britain, and Norway. Twenty-nine studies [6-8,12,19-21,25-27,30-36,38-47,49,51,52] used interviews, six were case studies [14,22,28,29,45,53], one used interviews and focus groups [9], and eight [10,11,13,23,24,37,48,50] used interviews supported by a questionnaire. For the eight studies that used both interviews and questionnaires, we relied on the qualitative results generated from the interviews.

### Narrative synthesis

We found that majority of studies had participants initiating use before they were 30 years old. In addition, histories of negative body image, psychological disorders such as mood and depressive disorders, and participation in power sports preceded initiation of AAS use for most persons. We also found that sources of first AAS were mainly users’ immediate social networks and the illicit market. Furthermore, we found that motives for AAS use were mainly enhanced sports performance, appearance, and muscle or strength.

### Age of AAS use initiation

Of the 24 studies that presented the ages at which participants initiated AAS use, initiation ages ranged from 14 to 54 years. However, only 5 of the 24 studies presented participants that initiated AAS use after age 30 consistent with evidence that about 80% of AAS users initiate use before age 30 [56]. It must be noted that some studies did not specify the ages at which some or all respondents initiated AAS use (See Table 3).

**Table 3:** Qualitative studies presenting age(s) of AAS use initiation

**Age(s) of initiation**	**Studies (first author, reference)**
14 years	Copeland [23] and Peters [24]; Kusserow [32]; Pope [45]; Tallon [11]
15 years	Copeland [23] and Peters [24]; Grogan [26]; Kusserow [32]; Skårberg [48,50]; Tallon [11]
16 years	Annitto [19]; Copeland [23] and Peters [24]; Grogan [26]; Korkia [12]; Skårberg [49]; Kimergård [8]; Joubert [7]; Tallon [11]
17 years	Copeland [23] and Peters [24]; Fudala [25]; Kusserow [32]; Tallon [11]
18 years	Copeland [23] and Peters [24]; Grogan [26]; Korkia [12]; Kusserow [32]; Pope [44]; Scull [47]; Joubert [7]; Tallon [11]
19 years	Boyadjiev [22]; Copeland [23] and Peters [24]; Grogan [26]; Korkia [13]; Pope [44]; Joubert [7]; Tallon [11]
20 years	Copeland [23] and Peters [24]; Cornford [9]; Grogan [26]; Pope [43]; Skårberg [49]; Tallon [11]
21 years	Copeland [23] and Peters [24]; Cornford [9]; Fudala [25]; Grogan [26]; Katz [29]; Pope [43]; Skårberg [49]; Tallon [11]
22 years	Copeland [23] and Peters [24]; Cornford [9]; Hegazy [28]
23 years	Copeland [23] and Peters [24]; Cornford [9]; Grogan [26]; Korkia [13]; Olrich [40]; Tallon [11]
24 years	Copeland [23] and Peters [24]; Cornford [9]; Fudala [25]; Malone [33]; Maycock [34,35]; Pope [44]; Joubert [7]; Tallon [11]
25 years	Copeland [23] and Peters [24]; Cornford [9]; Maycock [34,35]; Tallon [11]
26 years	Copeland [23] and Peters [24]; Cornford [9]; Fudala [25]; Tallon [11]
27 years	Copeland [23] and Peters [24]; Cornford [9]; Fudala [25]; Tallon [11]
28 years	Copeland [23] and Peters [24]; Cornford [9]; Skårberg [48,50]; Tallon [11]
29 years	Copeland [23] and Peters [24]; Cornford [9]; Grogan [26]; Tallon [11]
30 years	Copeland [23] and Peters [24]; Cornford [9]; Pope [43]; Tallon [11]
31 to 54 years	Copeland [23] and Peters [24]; Cornford [9]; Korkia [12]; Rashid [14]; Tallon [11]
Not specified	Bardick [20]; Bilard [21]; Fudala [25]; Gruber [27]; Joubert [7]; Katz [29]; Khorrami [30]; Kimergård [6,8]; Klötz [31]; Korkia [12,13]; Kusserow [32]; Maycock [34,35]; McKillop [36]; Midgley [37]; Nøkleby [38]; O’Sullivan [39]; Olrich [40]; Petrocelli [42]; Schwingel [46]; Scull [47]; Skårberg [48,50]; Tallon [11]; Todd [51]; Vassalo [52]; Walker [10]; Wilson-Fearon [53]

Not specified: Authors did not present age(s) of initiation for some or all participants.

### Pre-initiation history

Prior to initiating AAS use, participants had diverse backgrounds including sports (particularly power sports) participation, maladaptive relationships, psychopathology, negative self and body image, deviant behaviour, and abuse of other drugs (See Table 4).

**Table 4:** Qualitative studies presenting AAS users’ history prior to use

**History**	**Studies (first author, reference)**
Anorexia and reverse anorexia	Fudala [25]; Pope [44]
Complacent trainer(s)	Maycock [34,35]
Long-term exposure to muscle magazines	Petrocelli [42]
Low self-efficacy	Fudala [25]; Joubert [7]
Male sex work	Scull [47]
Negative body image	Fudala [25]; Khorrami [30]; Kusserow [32]; Maycock [34,35]; Petrocelli [42]; Pope [44]; Walker [10]; Joubert [7]
Other drug(s) use	Gruber [27]; Hegazy [28]; Joubert [7]; Kusserow [32]; Nøkleby [38]; Rashid [14]
Other sports (athletics, cycling, hockey, football etc.)	Bardick [20]; Boyadjiev [22]; Fudala [25]; Joubert [7]; Khorrami [30]; Kusserow [32]; Nøkleby [38]; Pappa [41]; Skårberg [49]; Tallon [11]; Vassalo [52]
Poor self-esteem	Fudala [25]; Walker [10]; Joubert [7]
Power sports (bodybuilding, powerlifting, weightlifting)	Annitto [19]; Bardick [20]; Bilard [21]; Boyadjiev [22]; Fudala [25]; Grogan [26]; Joubert [7]; Katz [29]; Khorrami [30]; Kimergård [8]; Korkia [12]; Kusserow [32]; Malone [33]; Maycock [34,35]; Olrich [40]; Petrocelli [42]; Pope [43]; Pope [44,45]; Skårberg [49]; Tallon [11]; Todd [51]; Wilson-Fearon [53]
Psychological disorder	Fudala [25]; Gruber [27]; Hegazy [28]; Pope [44,45]; Rashid [14]
Troubled background (bullying, divorce, rape etc.)	Fudala [25]; Gruber [27]; Hegazy [28]; Rashid [14]; Skårberg [48-50]; Joubert [7]

The most prominent feature of AAS users prior to initiation of use was participation in power sports such as bodybuilding, powerlifting, and weightlifting. This emerged in 23 studies [11-14,17,18,21,22,24-27,32,34-37,41,43,45,50,52,55]. It emerged in Maycock and Howat’s study [34] that users:

…had been weight training for three years prior to initiating anabolic steroid use. However, 11 of the interviewed subjects initiated use within one year of starting weight training (p. 319).

Similarly, participation in other sports such as athletics, cycling, hockey, and football emerged as a prominent feature of AAS users backgrounds prior to initiation of AAS use [7,11,22,25,30,32,38,41,49,52]. This is exemplified by Josh in Bardick et al.’s study [20]. Josh was a hockey player who “needed to take steroids to become the best” (p. 138). Similarly, Maycock and Howat [34] highlighted association with ‘complacent’ trainers or coaches as a feature of AAS users prior to the initiation of AAS use (p. 319).

Also, Gruber and Pope [27] recount the story of Ms. A. who “took all of the supplements and ergogenic drugs that her trainer recommended, including large doses of anabolic steroids”. In Maycock and Howat’s study [34]:

Four of the interviewed sample indicated that complacency by trainers and coaches contributed to their decision to consider use. The failure of coaches and officials to investigate large increases in body mass and strength achieved by other competitors contributed to their decision to explore use (p. 319).

AAS users also showed psychological syndromes such as mood and depressive disorders as well as troubled psychosocial histories including divorce, having suffered rape, poor parental connectedness or involvement, and poor social support [14,25,27,28,44,45,48-50] prior to the initiation of AAS use. In one study [27], five females initiated AAS use after the experience of rape:

None used such drugs previously…Indeed, prior to experiencing rape, these five women believed that taking anabolic substances was a weakness…Subsequent to their rape, they justified the decision to start using anabolic substances as being necessary to gain muscle mass and strength, because they thought it was impossible to grow big or strong enough “naturally” (p. 275).

Also evident as a feature of AAS users prior to initiation of AAS use was eating disorders such as anorexia nervosa [25,44]. Pope et al. [44] present the cases of four persons who initiated AAS use due to anorexia nervosa and reverse anorexia nervosa. Negative body image as well as low self-esteem and low self-efficacy also emerged as features of AAS users prior to the initiation of AAS use [7,10,25,30,32,34,35,42,44]. Cases 01 and 02 of Fudala et al.’s study [25] recount the stories of a male who “stated that he was using AASs because he lacked self-esteem and was not good-looking.” and another who initiated AAS use because he “felt small and [needed to] become more muscular to accomplish [his] goals” (p. 123).

Use of other drugs also emerged as a feature of AAS users prior to the initiation of AAS use [7,14,27,28,32,38]. Nøkleby and Skårderud [38] highlighted drug use networks as well as addiction clinics as major gateways for the initiation of use. In their study, Kristian commented:

I have always been offered steroids at other places as well, but it never came to anything. But when I got here [addiction clinic] it (steroids) fell right in my lap. And it was the same the last place I was in treatment. It (steroids) fell right in my lap, and that made it easy to accept (p. 495).

It also emerged that many AAS users understood the debilitating consequences of AAS but nevertheless went ahead to initiate use [10,32,34,40]. In Maycock and Howat’s study [34]:

Prior to initiating [AAS] use all of the men interviewed undertook information searches. These included talking to friends, gym trainers and instructors, anabolic steroid users and dealers, reading magazines, underground anabolic steroid manuals and medical journals and occasionally talking to medical practitioners (p. 320).

### Sources of first AAS

Studies specified several sources of users’ first AAS: the illicit market (dealers, mail order, internet etc.), coaches or trainers, clinicians or health workers (doctors, pharmacists, and veterinarians), friends or teammates, gym employees, intimate partners, and relatives (See Table 5).

**Table 5:** Qualitative studies presenting AAS users’ first sources of AAS

**Source**	**Studies (first author, reference)**
Coach/trainer	Copeland [23] and Peters [24]; Gruber [27]; Korkia [12,13]; Kusserow [32]
Doctor	Copeland [23] and Peters [24]; Korkia [12]; Kusserow [32]; O’Sullivan [39]
Friend/teammate	Bilard [21]; Copeland [23] and Peters [24]; Fudala [25]; Hegazy [28]; Kimergård [6]; Korkia [12]; Kusserow [32]; Nøkleby [38]; O’Sullivan [39]; Petrocelli [42]; Schwingel [46]; Skårberg [49]; Tallon [11]
Gym employee	Copeland [23] and Peters [24]; Khorrami [30]; Korkia [12,13]; Kusserow [32]; Walker [10]
Illicit market (dealers, internet)	Annitto [19]; Bilard [21]; Copeland [23] and Peters [24]; Kimergård [8]; Korkia [12]; Kusserow [32]; Maycock [34,35]; O’Sullivan [39]; Petrocelli [42]; Schwingel [46]; Todd [51]; Walker [10]
Intimate partner	Korkia [13]; Skårberg [49]
Pharmacist	Copeland [23] and Peters [24]; Kusserow [32]
Relative	Bilard [21]; Copeland [23] and Peters [24]; Fudala [25]
Veterinarian	Copeland [23] and Peters [24]; Kusserow [32]

The illicit market emerged as a major source of AAS during the initiation of AAS use [8,10,12,19,21,23,24,32,34,35,39,42,46,51]. The immediate social networks of respondents such as intimate partners, relatives, as well as friends or teammates also emerged as important sources of AAS [6,11,21,23-25,28,32,38,39,42,46,49] during the initiation of AAS use.

In addition, training associates such as coaches or trainers and gym employees emerged as a source of AAS during the initiation of AAS use [15,16,19,22,24,49,55,56]. Clinicians or health workers such as doctors, pharmacists, and veterinarians also came up as sources of AAS during the initiation of AAS use [23,24,32,39].

In a 1990 study of 72 current and former users [32], the sources of AAS were: friends/teammates (*n* = 41), pharmacists (*n* = 22), dealers (*n* = 17), veterinarians (*n* = 10), gym employees (*n* = 8), doctors (*n* = 3), and coach/team doctor (*n* = 1). Moreover, in a 1997 study [24], the sources of AAS were: friends (*n* = 64), doctors (*n* = 42), dealers (*n* = 41), pharmacists (*n* = 18), gym employees (*n* = 14), coaches/trainers (*n* = 14), veterinarians (*n* = 11), relatives (*n* = 6), mail order (*n* = 4), and other (*n* = 4). It is however worthy of note that in the most recent qualitative studies presenting sources of AAS [6,10,21,38,46], the only sources of AAS were the illicit market, relatives, and friends.

### Motives/drives for initiating AAS use

Motives for initiating AAS use were for: aggression, enhanced appearance, securing sports scholarships, enhanced muscle or strength, occupational (non-sporting) activities, personal security, psychological well-being or satisfaction, physiological recovery or injury prevention, sexual attraction, and for sporting or competitive activities. Other drives were trainers’ approval, curiosity, family influence, use by famous athletes portrayed in the media, peer influence, and use of AAS as a sport or social norm (See Table 6).

**Table 6:** Qualitative studies presenting AAS users’ motives/drives for initiating AAS use

**Motive/drive**	**Studies (first author, reference)**
Aggression	Klötz [31]; Kusserow [32]; Maycock [34,35]; Mckillop [36]
Appearance/body image	Annitto [19]; Bardick [20]; Bilard [21]; Copeland [23] and Peters [24]; Fudala [25]; Grogan [26]; Gruber [27]; Hegazy [28]; Khorrami [30]; Kimergård [6,8]; Klötz [31]; Kusserow [32]; Malone [33]; Maycock [34,35]; Midgley [37]; Nøkleby [38]; O’Sullivan [39]; Olrich [40]; Pappa [41]; Petrocelli [42]; Pope [44,45]; Rashid [14]; Schwingel [46]; Scull [47]; Skårberg [48-50]; Tallon [11]; Walker [10]
Coach’s/trainer’s approval/influence	Kusserow [32]; Maycock [34,35]
Curiosity	Olrich [40]; Pappa [41]; Skårberg [49]
Family influence	Khorrami [30]; Kusserow [32]; Joubert [7]
Media	Bardick [20]; Grogan [26]; Kusserow [32]; Pappa [41]; Walker [10]
Muscle/strength	Bilard [21]; Copeland [23] and Peters [24]; Cornford [9]; Fudala [25]; Gruber [27]; Hegazy [28]; Joubert [7]; Khorrami [30]; Kimergård [6]; Klötz [31]; Korkia [12,13]; Malone [33]; McKillop [36]; Midgley [37]; Nøkleby [38]; O’Sullivan [39]; Pappa [41]; Petrocelli [42]; Pope [45]; Rashid [14]; Schwingel [46]; Scull [47]; Skårberg [49]; Skårberg [48,50]; Tallon [11]; Walker [10]
Occupational (non-sporting)	Grogan [26]; Kimergård [6]; Maycock [35]; Olrich [40]; Schwingel [46]; Scull [47]
Peer influence	Joubert [7]; Kusserow [32]; Maycock [34,35]; Midgley [37]; Olrich [40]
Personal security	Bardick [20]; Cornford [9]; Gruber [27]; Joubert [7]
Physiological recovery/injury prevention	Bardick [20]; Bilard [21]; Copeland [23] and Peters [24]; Cornford [9]; Hegazy [28]; Korkia [12]; Kusserow [32]; Malone [33]; McKillop [36]; Midgley [37]; Tallon [11]
Psychological (well-being, self-esteem, self-efficacy, concentration, confidence)	Bardick [20]; Bilard [21]; Fudala [25]; Joubert [7]; Klötz [31]; Midgley [37]; Nøkleby [38]; Olrich [40]; Petrocelli [42]; Pope [45]; Rashid [14]; Tallon [11]; Walker [10]
Sexual attraction/attractiveness	Kusserow [32]; Olrich [40]; Petrocelli [42]; Tallon [11]
Sport/social norm	Bilard [21]; Grogan [26]; Klötz [31]; Malone [33]; Olrich [40]; Pappa [41]; Skårberg [49]; Todd [51]; Kimergård [8]
Sports	Bardick [20]; Bilard [21]; Boyadjiev [22]; Copeland [23] and Peters [24]; Fudala [25]; Grogan [26]; Joubert [7]; Katz [29]; Khorrami [30]; Klötz [31]; Korkia [12,13]; Kimergård [6]; Kusserow [32]; Malone [33]; Maycock [34,35]; McKillop [36]; Midgley [37]; Nøkleby [38]; Olrich [40]; Pappa [41]; Pope [43]; Schwingel [46]; Skårberg [48-50]; Todd [51]; Wilson-Fearon [53]
Sports scholarship	Kusserow [32]; Vassalo [52]

Of the above motives and drives, initiation of AAS use for enhanced appearance or body image, muscle or strength, and sports or athletic performance were most prominent in the literature. Indeed, in a study of Australian AAS users [24], the most paramount motives for the initiation of AAS use were improved appearance (46%), increase in size (33%), increase in strength (7%), and improved sporting performance (6%). Case 04 of Fudala et al.’s study [25] also tells the story of a 22-year-old male who initiated AAS use at the age of 17 “in order to increase his size and power for football” and consecutively increased his AAS consumption “in order to compete in bodybuilding events”. Paula, a 39-year-old affirms the relationship between her AAS use and sports participation in Grogan et al.’s study [26] with the confession “I will stop [using steroids] when I stop competing yeah” (p. 853). Similarly, others initiated AAS use for physiological recovery or injury prevention [9,11,12,20,21,23,24,28,32,33,36,37].

Related to enhanced sports performance, enhanced occupational functioning also emerged as motive for the initiation of AAS use [6,26,34,35,40,46,47]. In support of this motive, Matt, a 33-year-old male stripper commented in Scull’s study [47]: “All the guys [male strippers] take steroids, you know?…See, you won’t last long in this industry if you don’t use steroids. They all do steroids” (p. 567). Improved occupational functioning was again highlighted in Maycock and Howat’s study [35]:

For the doormen and security workers, it was about projecting physical competence; for the power lifters, it was about projecting the image of brute strength; for the sex workers or gay men using for body image reasons, it was about the presentation of a natural healthy look. For bodybuilders, it was about projecting their muscles, size and shape (p. 861).

Sexual attraction or attractiveness also emerged as an important motive for the initiation of AAS use [11,32,40,42]. This is highlighted by Kusserow’s [32] finding that 18% of AAS users initiated use in order to “be more successful with the opposite sex” (p. 7). In addition, Petrocelli et al. [42] indicated that AAS use:

increased and enhanced [users’] confidence and love life, as they claimed having a defined, muscular physique allowed them to meet and have sexual relations with more partners (p. 1194).

Social pressure in the form of media influence, peer influence, and sport or social norms also emerged as an important drive for the initiation of AAS use. Related to this, Petrocelli et al. [42] found long-term exposure to muscle magazines as a feature of AAS users prior to initiation of AAS use. In addition, Joe a 29-year-old male commented: “I came from a solid family that stressed competition and giving it 110%. So when I didn’t see the results in the gym, I went to steroids” [22, p. 10]. In Grogan et al.’s study [26], John, a 25-year-old indicated:

The more I trained, the more magazines I looked at, the bigger I wanted to be. …and there was an ITV programme [about body builders] and when I watched these people it made me feel really depressed. I didn’t look as good as them. And it had a massive effect on my decision to take steroids. In fact it was probably one of the biggest reasons why I did take them seeing other people bigger than me (p. 853).

There is however contrary evidence of the influence of media on AAS use. In Walker and Joubert’s study [10], 66% of respondents stated that the media had no influence on their desire to use AAS although these respondents believed that most muscular men portrayed in the media use AAS.

Moreover, psychological well-being emerged as an important motive for the initiation of AAS use [7,10,11,14,20,21,25,31,37,38,40,42,45]. Specific psychological motives for initiating use included boosting self-esteem, confidence, concentration, and overcoming psychological disorders such as depression.

It is important to note however that motives for AAS use may change with time. For instance, in an Australian study [24], 46% of users indicated that they initiated use in order to improve their appearance. However, only 35% of these respondents mentioned improved appearance as motive for their most recent use indicating motive change in some users after initiation. Disparities were also discovered for other motives (p. 37). A security worker also elucidated motive change in a recent study by Kimergård [6]:

At this moment in time, I’m not looking to get any bigger as a bodybuilder for example. I like to increase my strength, and now it’s more for conditioning…My next cycle, I’ll be doing a ‘cutting’ cycle, I’ll be dieting and getting down to a reasonable healthy weight (p. 3).

### Implications for research

The results of our study have important implications for future investigations. First, unnecessary replication of qualitative research may be avoided when systematic reviews and qualitative syntheses are conducted prior to the execution of new qualitative research. In addition, all studies were conducted in Western countries. This is problematic as there is evidence that nonmedical AAS use represents a global public health problem [1]. Thus, future studies must as well endeavour to investigate the experiences of AAS users in non-Western countries.

Our findings also reveal a relative paucity of qualitative investigations on the influence of backgrounds of anorexia nervosa, complacent trainers, use of other appearance and performance enhancing drugs and methods, long-term exposure to media images of muscular persons, low self-esteem and self-efficacy, and male sex work on the initiation of AAS use. Moreover, scant qualitative studies have examined the influence of motives and drives such as securing sports scholarships, coaches’ or trainers’ approval, the search for confidence, curiosity, the influence of famous athletes, family influence, and personal security on the initiation of AAS use. Thus, future studies should examine these topics.

### Implications for policy and practice

Arguably, our findings represent an important basis for policymaking and planning. First, with evidence from the present study that most AAS users initiate use under 30 years, AAS use interventions should focus primarily on adolescents and young adults. Thus preventive interventions should be tailored mainly for these age cohorts. In addition, with evidence from our study that negative body image, psychological disorders, and sports participation (particularly in power sports) precede initiation of AAS use for most persons, AAS use interventions must target persons demonstrating these characteristics as well as focus on relevant environments.

Moreover, AAS use interventions must be targeted at individuals with: eating disorders, low self-esteem and self-efficacy, ‘doping-complacent’ trainers, long-term exposure to media images of muscular persons, troubled backgrounds, drug use histories and milieus, and psychological disorders. AAS use interventions should also be aimed at athletes especially power sportspeople, doormen and security workers, male sex workers, and gay men as these groups emerged as popular AAS users in this qualitative metasynthesis.

Again, it is worrying that although some AAS users appreciated the debilitating consequences of AAS, they nevertheless went ahead to initiate use [6,10,32,34,40]. We also found that sources of first AAS were mainly users’ immediate social networks and the illicit market. Furthermore, it is worthy of note that in the most recent qualitative studies presenting sources of AAS [6,10,21,38,46], the only sources were the illicit market, relatives, and friends. This is perhaps attributable to the increasing illegalization of AAS use since the 1990s [1]. Nevertheless, with the proliferation of both legal and illegal substances on the illicit market and the internet, as well as the expectedly ‘drug-clean’ environments of addiction clinics [38], better ways of dealing with the global public health problem of AAS use will need to be found.

### Strengths and weaknesses

The present study has several strengths. To our knowledge, it is the first-ever systematic review and synthesis of qualitative studies on AAS use initiation. The systematic and advanced strategy for identifying, reporting, and synthesizing qualitative studies, the ‘global’ and comprehensive nature of the present study, and the inclusion of a large number of both peer-reviewed and grey literature are also notable assets.

Despite the aforementioned strengths of the present study, some limitations ought to be noted when interpreting our results. First, we restricted our analysis to English language literature. Though this is not an uncommon practice for systematic reviews [57], it is possible that the exclusion of non-English language literature influenced our results. However, it must be noted that Moher et al. [57] found no evidence of biased results with the exclusion of non-English studies. Nevertheless, it is worth pointing out again that our themes and results were robust in the sensitivity analysis. Furthermore, it is plausible that the case studies included in the present study were reported due to their ‘unusual’ or ‘exceptional’ nature. Thus, these cases may not be representative of the typical AAS user.

## Conclusions

Arguably, our findings represent an important basis for AAS use interventions. Findings from the present study denote the importance of psychological and social factors in the initiation of AAS use. Our findings also complement available evidence from quantitative studies on the initiation of AAS use. There is the need for improved ways of dealing with the global problem of AAS use with the increased availability of both legal and illegal substances on the illicit market and the internet.

## Competing interests

The authors declare that they have no competing interests.

## Authors’ contributions

DS led the conception and design of the study, the literature search, analysis, writing and revision of the manuscript. CSA and SP contributed to the writing and revision of the manuscript. All authors read and approved the final manuscript.

## Authors’ information

DS is a PhD research fellow at the Department of Psychosocial Science, University of Bergen, Norway. He conducts research on image and performance enhancing drugs and methods with special focus on anabolic-androgenic steroids. He also works on other drug and behavioural addictions. CSA is a postdoctoral research fellow at the Department of Psychosocial Science, University of Bergen, Norway, and a clinical psychologist at the Bergen Clinics Foundation, Norway. She conducts research in the area of work, industrial and organizational psychology, as well as drug and behavioural addictions. SP is a professor of psychology at the Department of Psychosocial Science, University of Bergen, Norway, and a senior researcher at the Norwegian Competence Centre for Sleep Disorders. He conducts research on sleep and sleep disorders as well as drug and behavioural addictions.
